# Supplementary material for: Transcriptome Analysis Reveals Vimentin-Induced Disruption of Cell–Cell Associations Augments Breast Cancer Cell Migration
Source: Cells. 2022 Dec 13;11(24):4035. doi: 10.3390/cells11244035 (PMC9776984; doi:10.3390/cells11244035)
Supplement: Supplementary file 1 [file cells-11-04035-s001.zip › DEGs and enrichment analysis.zip!/DEGs and enrichment analysis/WT_VIMvsVector GO enrichment/GSEA/WT_VIM/ASTROCYTE_DEVELOPMENT/ASTROCYTE_DEVELOPMENT(GO_0014002).html]

Details for gene set ASTROCYTE\_DEVELOPMENT(GO:0014002)[GSEA]


|  || Dataset | fpkm.sample |
| Phenotype | sample.cls |
| Upregulated in class | WT\_VIM |
| GeneSet | ASTROCYTE\_DEVELOPMENT(GO:0014002) |
| Enrichment Score (ES) | 0.9393729 |
| Normalized Enrichment Score (NES) | 1.4819343 |
| Nominal p-value | 0.0 |
| FDR q-value | 0.16300026 |
| FWER p-Value | 0.163 |
Table: GSEA Results Summary

  

Fig 1: Enrichment plot: ASTROCYTE\_DEVELOPMENT(GO:0014002)
  
Profile of the Running ES Score & Positions of GeneSet Members on the Rank Ordered List

  

| PROBE | DESCRIPTION   (from dataset) | GENE SYMBOL | GENE\_TITLE | RANK IN GENE LIST | RANK METRIC SCORE | RUNNING ES | CORE ENRICHMENT || 1 | ENSG00000087250 | MT3 |  |  | 1710 | Infinity | -0.0289 | Yes |
| 2 | ENSG00000026025 | VIM |  |  | 2388 | 1709.539 | 0.9394 | Yes |
| 3 | ENSG00000050555 | LAMC3 |  |  | 3283 | 4.008 | 0.9265 | No |
| 4 | ENSG00000186868 | MAPT |  |  | 3555 | 3.590 | 0.9240 | No |
| 5 | ENSG00000169071 | ROR2 |  |  | 3924 | 3.097 | 0.9196 | No |
| 6 | ENSG00000184486 | POU3F2 |  |  | 4174 | 2.884 | 0.9170 | No |
| 7 | ENSG00000146216 | TTBK1 |  |  | 4279 | 2.799 | 0.9168 | No |
| 8 | ENSG00000130164 | LDLR |  |  | 5405 | 2.231 | 0.8991 | No |
| 9 | ENSG00000123384 | LRP1 |  |  | 7159 | 1.782 | 0.8705 | No |
| 10 | ENSG00000172037 | LAMB2 |  |  | 7355 | 1.746 | 0.8682 | No |
| 11 | ENSG00000204305 | AGER |  |  | 8632 | 1.530 | 0.8474 | No |
| 12 | ENSG00000145191 | EIF2B5 |  |  | 9494 | 1.418 | 0.8337 | No |
| 13 | ENSG00000131095 | GFAP |  |  | 14245 | 1.007 | 0.7539 | No |
| 14 | ENSG00000128271 | ADORA2A |  |  | 14382 | 0.996 | 0.7522 | No |
| 15 | ENSG00000142192 | APP |  |  | 15899 | 0.926 | 0.7271 | No |
| 16 | ENSG00000184845 | DRD1 |  |  | 16190 | 0.907 | 0.7227 | No |
| 17 | ENSG00000242689 | CNTF |  |  | 17037 | 0.855 | 0.7088 | No |
| 18 | ENSG00000196712 | NF1 |  |  | 17466 | 0.829 | 0.7021 | No |
| 19 | ENSG00000143546 | S100A8 |  |  | 18155 | 0.790 | 0.6909 | No |
| 20 | ENSG00000133703 | KRAS |  |  | 19512 | 0.715 | 0.6684 | No |
| 21 | ENSG00000105810 | CDK6 |  |  | 21736 | 0.593 | 0.6311 | No |
| 22 | ENSG00000080815 | PSEN1 |  |  | 22258 | 0.559 | 0.6226 | No |
| 23 | ENSG00000163220 | S100A9 |  |  | 23819 | 0.453 | 0.5965 | No |
| 24 | ENSG00000146648 | EGFR |  |  | 24583 | 0.392 | 0.5838 | No |
| 25 | ENSG00000123560 | PLP1 |  |  | 25259 | 0.324 | 0.5725 | No |
| 26 | ENSG00000185483 | ROR1 |  |  | 25633 | 0.291 | 0.5664 | No |
| 27 | ENSG00000128602 | SMO |  |  | 26169 | 0.234 | 0.5575 | No |
| 28 | ENSG00000136869 | TLR4 |  |  | 26317 | 0.214 | 0.5551 | No |
| 29 | ENSG00000134198 | TSPAN2 |  |  | 26909 | 0.129 | 0.5452 | No |
| 30 | ENSG00000171049 | FPR2 |  |  | 31331 | NaN | 0.4704 | No |
| 31 | ENSG00000198719 | DLL1 |  |  | 56017 | NaN | 0.0528 | No |
Table: GSEA details
[plain text format]

  

Fig 2: ASTROCYTE\_DEVELOPMENT(GO:0014002)
  
Blue-Pink O' Gram in the Space of the Analyzed GeneSet

  

Fig 3: ASTROCYTE\_DEVELOPMENT(GO:0014002): Random ES distribution
  
Gene set null distribution of ES for
 **ASTROCYTE\_DEVELOPMENT(GO:0014002)**
